# Effect of Potato Dietary Fiber on the Quality, Microstructure, and Thermal Stability of Chicken Patty

**DOI:** 10.3390/foods11243978

**Published:** 2022-12-08

**Authors:** Jia Feng, Baohua Kong, Fangda Sun, Xiufang Xia

**Affiliations:** College of Food Science, Northeast Agricultural University, Harbin 150030, China

**Keywords:** dimensional change, water- and fat-binding properties, moisture migration, sensory properties

## Abstract

A total of 150 chicken patties containing different concentrations of potato dietary fiber (PDF) (0.0–4.0%) (30 for every treatment) with three replicates were used to access the influence of PDF on their quality, microstructure, and thermal stability. PDF improved the quality of chicken patty, including significantly inhibiting dimensional change and improving water- and fat-binding properties and textural properties (*p* < 0.05). Moreover, PDF promoted a more homogeneous and dense meat–protein network structure to be formed. The results of thermal stability showed that PDF did not affect the thermal denaturation of proteins (*p* > 0.05). The samples with PDF (<3.0%) did not have a significant negative effect on sensory properties of chicken patty; meanwhile, there were more abundant nutrients and a lower energy value in samples with PDF compared with the control. Therefore, PDF could be a promising ingredient to improve the properties of chicken patties, which was related to the amount of PDF added and performed best at 3.0% level.

## 1. Introduction

Patties, a prefabricated meat product that meets the needs of a fast-paced lifestyle [1], are the central ingredient in burgers. This product is usually frozen or refrigerated before being thawed and cooked in fast-food restaurants, and this processing can cause significant shrinkage in the patties. This phenomenon is caused primarily by water and fat loss which is induced by protein shrinkage caused by protein denaturation during heating. It also leads to other quality degradation in patties, such as a decrease in yield [2]. These changes will lead to financial losses, reduce the reputation of the company, and reduce consumer acceptability. This problem has been brought to the attention of companies and consumers.

Chemicals (phosphates) and plant-based ingredients such as seed flour [3], destoned olive cake powder [4], and dietary fiber [5] are often used to address patty shrinkage during processing. However, the chemicals can cause an increased risk of cardiovascular disease, inhibit calcium absorption [6], and cause other factors detrimental to human health, so plant-based ingredients are increasingly preferred to inhibit patty shrinkage during processing. Compared with other plant ingredients, dietary fiber has high water- and oil-holding capacity, which can be used as an effective binder and filler [7]. Furthermore, it has positive implications for improving other quality characteristics of meat products, such as emulsification stability, textural properties, and gel-forming capacity [8]. More importantly, dietary fiber has specific functional and nutritional properties, such as decreasing the incidence of chronic diseases including colon cancer, diabetes, and gastrointestinal cancer [9].

Dietary fiber can be extracted from fruits, vegetables, crops, and processing by-products such as potato peel. Potato peel is a waste product of potato starch production and will cause significant environmental pollution if not handled properly [10]. Potato peels are widely available and rich in dietary fiber (higher than wheat bran) and are an excellent source for extracting high water retention dietary fiber [11]. The current applications of potato dietary fiber (PDF) are focused primarily on the texture, water distribution, thermal, and gluten properties of frozen dough during storage [12], and bread physicochemical characteristics during storage [13]. However, few researchers have considered the application of PDF to the dimensional change inhibition of meat products and the quality improvement of meat products.

Therefore, the purpose of this study is to evaluate the influence of PDF on quality, microstructure, and thermal stability of fried chicken patties. The ultimate aim is to investigate the potential technological use of PDF in improving the properties of processed meat products.

## 2. Materials and Methods

### 2.1. Materials and Chemicals

Fresh chicken breast muscle and pork back fat were obtained from a local supermarket (Harbin, China). PDF was provided by Kangzhou Biotechnology Co., Ltd. (Xi’an, China), the concentration of insoluble dietary fiber was 90%, and the particle size was 100-mesh (147 μm). The color of PDF was CIE L* = 88.3 ± 0.07; a* = 1.55 ± 0.23; b* = 9.59 ± 0.08. The water absorption capacity of PDF was 10.4 ± 0.1 g/g, the oil capacity was 2.51 ± 0.77 g/g, and the swelling capacity was 15.5 ± 0.1 mL/g, according to the method of Bender et al. [14].

### 2.2. Chicken Patties Preparation and Processing

**Figure 1:**
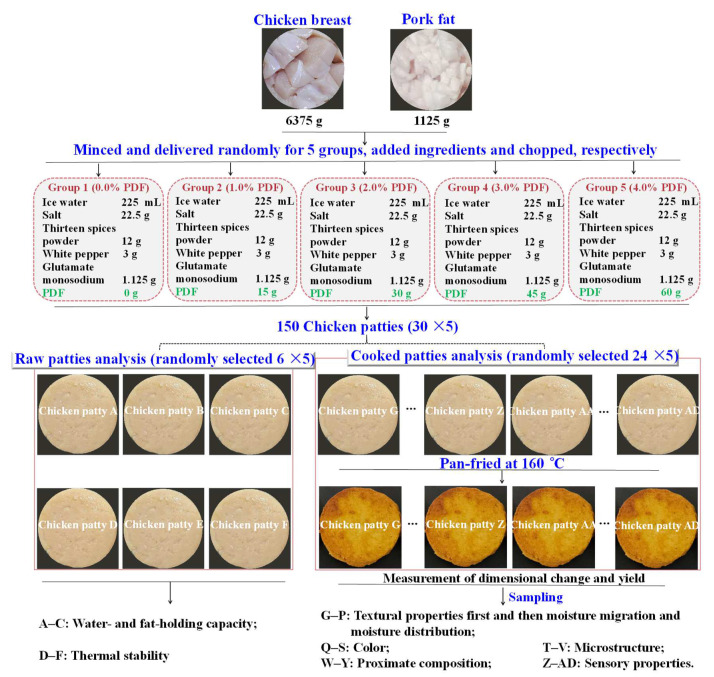
Distribution and measurement of samples.

Fresh chicken breast muscle and pork back fat were stored on ice and transported to the laboratory within 1 h. The preparation of chicken patty was processed according to the method of Pan et al. [15] with slight modifications. First, all visible connective tissue and fat were trimmed from the chicken breast. Then, meat and fat were minced separately using 4 and 3 mm plates in a BJRJ-82 meat grinder (Expro Industrial Co., Ltd., Jiaxing, China). For each group, chicken breast and pork back fat were added in a ratio of 85:15 and mixed with other ingredients according to Figure 1 for chopping. The chopping time for this experiment was 4 min, the entire chopping process was operated at 4 °C, and the final temperature of the patties was less than 12 °C. The distribution and measurement of samples are shown in Figure 1. All patties (10 patties for every replicate) for every treatment (approximately 58 g each) were manufactured via a round mold (the diameter and thickness were 6.80 and 1.60 cm, respectively). Finally, all of them were individually wrapped in polyethylene bags and reserved at 4 °C, then 6 patties were used for the analysis of water- and fat-binding properties and thermal stability; the other part of patties were pan-fried for determination of other indicators. Three independent batches of the samples were used, and each batch of the samples was performed three times.

The pan (diameter: 26 cm, JLW2801D, Joyoung Co., Ltd., Jinan City, China) was preheated for 2 min, and the chicken patties were pan-fried in 40 mL soybean oil at 160 °C and turned over every 30 s to avoid burning; the heating endpoint is a central temperature of 74 °C. The core temperature was measured using a digital probe thermometer (TP101, Funde Technology Beijing Co., Ltd., Beijing, China). The patties were placed in the same position in the pan, one sample at a time, and the oil was changed every three times throughout the frying process to minimize errors. After frying, the chicken patties were cooled to 20 °C for further analysis.

### 2.3. Quality

#### 2.3.1. Dimensional Change and Yield

The dimensional change of the chicken patties was measured according to the method described by [2] with slight modifications. A vernier caliper and electronic scale were used to measure the diameter, thickness, and yield of the chicken patties before frying and 1 h after frying. The diameter and thickness were measured for each sample at ten points spread evenly across the chicken patties; the mean value was recorded as the result of one replicate and took the average of three replicates as the final result of diameter and thickness, respectively. The reduction of diameter, increase of thickness, reduction of volume, and yield of the samples can be obtained via Equations (1)–(4):

(1) $$\mathrm{Reduction}\;\mathrm{of}\;\mathrm{diameter}\;\left(\%\right)=\frac{D_{b}-D_{a}}{D_{b}}\;\times \;100\%$$

(2) $$\mathrm{Increase}\;\mathrm{of}\;\mathrm{thickness}\;\left(\%\right)=\frac{T_{a}-T_{b}}{T_{b}}\;\times \;100\%$$

(3) $$\mathrm{Reduction}\;\mathrm{of}\;\mathrm{volume}\;\left(\%\right)=\frac{V_{b}-V_{a}}{V_{b}}\;\times \;100\%$$

(4) $$\mathrm{Yield}\;\left(\%\right)=\frac{W_{b}-W_{a}}{W_{b}}\;\times \;100\%$$

where *D*_b_, *T*_b_, *V*_b_, and *W*_b_ are the diameter, thickness, volume, and weight of the chicken patties before frying (cm/cm/cm^3^/g), and *D*_a_, *T*_a_, *V*_a_, and *W*_a_ are the diameter, thickness, volume, and weight of the chicken patties after frying (cm/cm/cm^3^/g).

#### 2.3.2. Water- and Fat-Holding Properties

The water- and fat-holding properties are expressed as total loss, water loss, and fat loss. Approximately 35 g of raw chicken patty was placed in 50 mL centrifuge tubes and centrifuged at 3500× *g* for 15 min to remove air bubbles. The tubes were heated in a 75 °C water bath for 30 min then immediately opened and left to stand upside down for 1 h to release the separated fat and water onto a plate and heated in a stove at 105 °C (SW-90D, Sang WooScienctific Co., Ltd., Pyeongtaek-si, Republic of Korea). The calculation of total loss, water loss, and fat loss were according to the method of Zhuang et al. [7].

#### 2.3.3. Moisture Migration

The moisture migration of fried chicken patty was evaluated using a MesoMR23–060H-I NMR analyzer (Niumag Co., Ltd., Suzhou, China). The samples were extracted from the central portion of each patty with a sampler (25 mm diameter), then removed the crust, adjusted the height to 10 mm, and placed in the sample tank. The transverse relaxation time (T2) was accounted using the Carr–Purcell–Meiboom–Gill pulse sequence and the CONTIN algorithm. After normalizing the raw data, the transverse relaxation time (T2) was measured using the MultiExp Inv Analysis V1.0 software (Niumag Co., Ltd., Shanghai, China).

#### 2.3.4. Moisture Distribution

After the moisture migration measurement was completed, the instrument continued to measure the moisture distribution by spin-echo (SE) imaging sequence. The parameters of the instrument were set as follows: field of view of 80.0 × 80.0 mm, slice width of 5.00 mm, slice gap of 0.50 mm, read size of 256, phase size of 192, repetition time (TR) of 1600 ms, and echo time (TE) of 20.0 ms. The image level was selected according to Larmor’s law, and the signal-noise ratio and image clarity were adjusted. The proton density maps were initially processed by Niumag NMR Imaging System V 3.0 software.

#### 2.3.5. Color

The surface color of fried chicken patties was measured according to the method of Pan et al. [16] and Li et al. [17] with a color difference meter (WSC-S, Suzhou Optics Instrument Co., Ltd., Suzhou, China). The standard white board ((L* = 95.26, a* = −0.89, b* = 1.18) was used to calibrate the instrument. Three different points on each patty were selected for color analysis. The *∆E* of the samples can be obtained via Equation (5):

(5) $$∆E=\sqrt{\left(L^{*},-,L_{0}^{*}\right){}^{2}+\left(a^{*},-,a_{0}^{*}\right){}^{2}+\left(b^{*},-,b_{0}^{*}\right){}^{2}}\;\times \;100\%$$

where $L_{0}^{*}$, $a_{0}^{*}$, and $b_{0}^{*}$ are the color parameters of the control sample, and $L^{*}$, $a^{*}$, and $b^{*}\;$ are color parameters of other samples.

#### 2.3.6. Texture Profile Analysis (TPA)

The textural properties of chicken patty were measured according to the method of Bai et al. [18] with some modifications. Two deformation test (TDT) was used to assess the texture properties of fried chicken patties via a TA-TX plusC texture analyzer (Stable Micro Systems Co., Ltd., Godalming, UK) with a P/50 cylindrical probe (50 mm diameter). A sampler (25 mm diameter) was used for extracting from the central portion of each sample; the adjusted sample height was 10.0 mm. The testing parameters were fixed as follows: pre-test speed of 2.0 mm/s, test speed of 1.0 mm/s, post-test speed of 5.0 mm/s, strain of 30.0%, time of 5.0 s, and trigger force of 5.0 g.

### 2.4. Microstructure

The microstructures of the fried chicken patties were observed using scanning electron microscopy (SEM). The pieces (2 × 5 mm) were obtained with a double-edge blade from the central point of chicken patties for the measurement of SEM and the pre-treatment of samples proceeded according to the method of Wu et al. [19], with a magnification of 1000×.

### 2.5. Thermal Stability

The thermal stability of chicken batter with different PDF was determined by a differential scanning calorimetry calorimeter (DSC) (250, TA Instruments, New Castle, DE, USA), according to the method of Du et al. [20] with slight modifications. Lean meat (11–14 mg) was accurately weighed into a standard aluminum pan hermetically sealed and then scanned from 20 to 100 °C at a heating rate of 3 °C/min.

### 2.6. Sensory Properties

Sixteen panelists (eight males and eight females) were selected from a pool of graduate students who were previously trained through three preliminary sessions for sample familiarization. Before sensory analysis, each fried chicken patty was cooked until the core temperature reached 74 °C and cooled to 20 °C, then each sample was cut into four quarters (2.0 cm length × 2.0 cm wide × 1 cm height) along the center and mixed the samples for every group. The samples randomly selected were individually offered to panelists in a randomized monadic order, coded with three random numbers, and presented in a balanced order. Sensory scores were rated using a seven-point linear scale: for the color of the chicken patty, 1 = undesirable and 7 = desirable; for flavor, 1 = undesirable and 7 = desirable; for juiciness, 1 = dry and 7 = juicy; for texture, 1 = deep soft or hardness and 7 = moderate hardness. As consumers, panelists were also asked for their opinion on the overall acceptability (1 = low and 7 = high) of the fried chicken patty.

### 2.7. Proximate Composition

The compositional properties of the fried chicken patties were determined using the method of AOAC [21]; the patties were cut, homogenized, and mixed as samples for analysis. Moisture content (950.46) was determined by weight loss after 12 h of drying at 105 °C in a drying oven (SW-90D, Sang WooScienctific Co., Ltd., Seongnam, Republic of Korea). Fat content (960.39) was determined using Soxhlet extraction method and protein content (988.05) was determined by the Kjeldahl method with an automatic Kjeldahl nitrogen analyzer. Ash was determined according to the [21] method 923.03 (muffle furnace). The carbohydrate content of patties was determined by calculating the percent remaining to 100% after all the other components were measured. Total calorie estimates (kcal) for fried chicken patties were calculated on the basis of a 100 g portion using Atwater values for fat (9 kcal/g), protein (4.02 kcal/g), and carbohydrate (3.87 kcal/g) [22].

### 2.8. Statistical Analysis

The experiments were performed at least three times and each sample was analyzed three times. All data were presented as mean ± standard error (SE) and analyzed using Duncan’s test and one-way ANOVA of IBM SPSS statistics 25.0 (Tulsa, OK, USA) at the significance level of *p* < 0.05 to determine the influence of PDF concentration on chicken patty (dimensional change and yield, water- and fat-holding properties, moisture migration, color, texture properties, thermal stability, sensory properties, and proximate composition). Figures are plotted by using SigmaPlot 12.5.

## 3. Results and Discussion

### 3.1. Quality

#### 3.1.1. Dimensional Change and Yield

**Figure 2:**
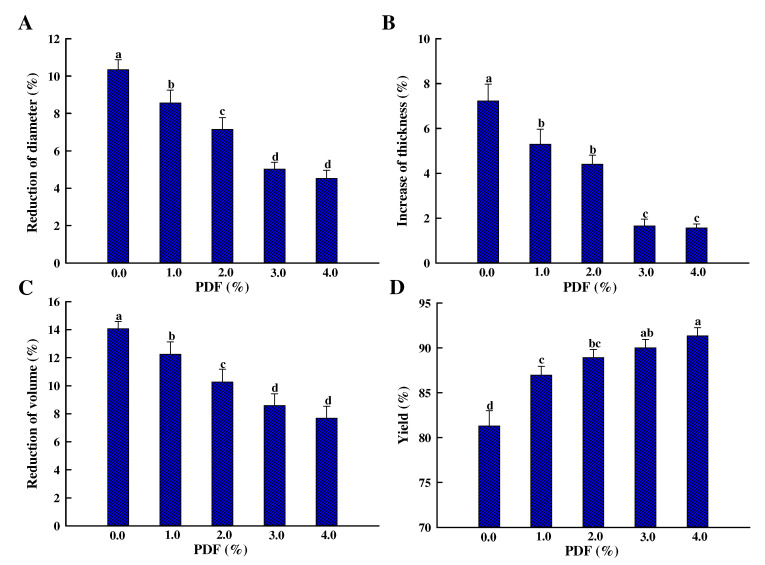
Influence of PDF on the dimensional change: (**A**) reduction of diameter; (**B**) increase of thickness; (**C**) reduction of volume; and (**D**) yield of chicken patty. Values represent mean ± SE of at least triplicate determinations. PDF: potato dietary fiber. The means at different amounts of PDF with different lowercase letters (a–d) differ significantly (*p* < 0.05).

The influence of PDF on the dimension (diameter, thickness, and volume) and yield of fried chicken patties are presented in Figure 2A–D. These qualities of patties were significantly changed after frying (*p* < 0.05); the diameter, volume, and weight decreased by 10.3%, 14.1%, and 18.7%, and the thickness increased by 7.23%. Sánchez-Zapata et al. [5] also observed that the diameter decreased by 15.6% and thickness increased by 10.2% in pork burgers after cooking in a convection oven. The reduction in patty diameter, volume, and weight is chiefly induced by water and fat loss caused by muscle protein denaturation and shrinkage during frying [23]. During the frying process, there is a temperature difference between the upper (~70 °C) and lower (~160 °C) patty layers. Furthermore, the water evaporation of the lower patties pushed the upper patties upward via expansion, which led to a radial expansion of the patties [24].

Furthermore, the diameter, volume, and weight of fried patties increased, and the thickness decreased as PDF increased. There were no significant differences in these indicators in patties containing 3.0% and 4.0% PDF (*p* > 0.05). The diameter, volume, and weight of fried patties with 3.0% PDF decreased by 5.03%, 8.58%, and 9.99%, while the thickness increased by 1.65% after heating. The dietary fiber with binding and stabilizing properties could form a stable system and prevent the loss of water and fat from patties [5], increasing patty diameter, volume, and weight. Zhuang et al. [25] reported that the porous and dendritic structure of the dietary fiber could bind water and fill in the meat–protein matrix, which may also contribute to increasing patty diameter. The changes in patty thickness with PDF were controlled because water evaporation decreased during frying.

#### 3.1.2. Water- and Fat-Holding Properties

**Figure 3:**
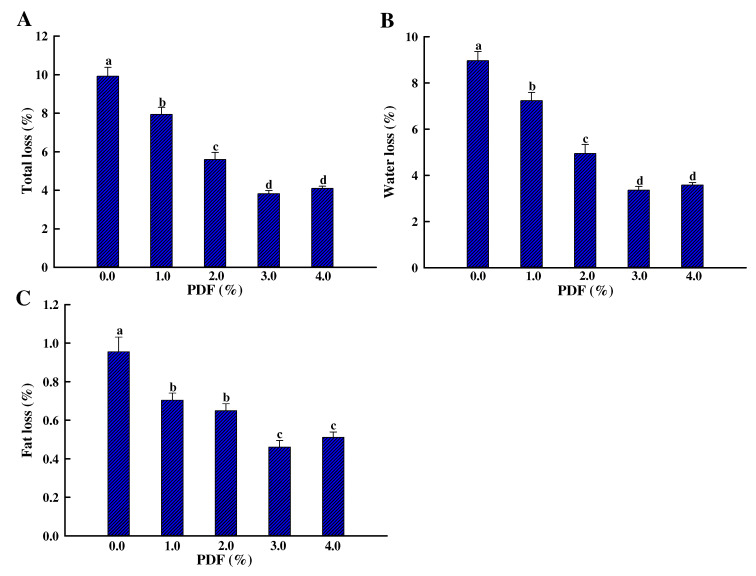
Influence of PDF on the water- and fat-holding properties ((**A**) total loss; (**B**) water loss; (**C**) fat loss) of chicken patty. Values represent mean ± SE of at least triplicate determinations. PDF: potato dietary fiber. The means at different amounts of PDF with different lowercase letters (a–d) differ significantly (*p* < 0.05).

The water- and fat-holding properties of chicken patties are expressed as total released, as presented in Figure 3A. There were significant differences in this parameter among the patties with or without PDF (*p* < 0.05), where water loss and fat loss of sample with 3.0% PDF decreased by 62.6% and 52.1% compared with the control (Figure 3B,C). These results imply that PDF can retain the water in meat products and reduce fat loss. Zhuang et al. [7] also reported that these three parameters of meat batter with 3.0% sugarcane dietary fiber decreased by 64.6%, 65.5%, and 43.8% compared with the control.

PDF, as an active dehydrating agent, could decrease water loss by trapping water in patties because of its absorbing and binding water capacity. The fat released was also lower in patties with PDF than without PDF because of its oil-binding properties. Therefore, the dimensional change and weight loss of chicken patties were also inhibited by water and fat retention of PDF (Figure 2).

#### 3.1.3. Moisture Migration

The moisture migration of the chicken patty is characterized by *T*_2*b*_, *T*_21_, and *T*_22,_ and *P*_2*b*_, *P*_21_, and *P*_22_ using low-field nuclear magnetic resonance (LF-NMR), a fast, non-destructive technique [26,27]. The changes in *T*_2*b*_, *T*_21_, and *T*_22_, and *P*_2*b*_, *P*_21_, and *P*_22_ of the chicken patty with different PDF content are presented in Table 1.

*T*_2*b*_ was not significantly influenced by PDF (*p* > 0.05) because this fraction of water is attached to protein primarily by covalent bonds and is not changed easily [8]. In contrast, both *T*_21_ and *T*_22_ shortened significantly with the increase in PDF (*p* < 0.05). These results demonstrate that these two types of water bind more tightly to large molecules such as proteins, and their mobility diminishes. The reduction in *T*_21_ may be explained by the water-stabilizing property of PDF. Furthermore, PDF with polar groups can absorb some of the free water, decreasing *T*_22_. The change in relaxation time may be associated with the dense degree of microstructure, protein aggregation, and water-holding capacity [28].

There was no significant difference in *P*_2*b*_ among all treatment groups (*p* > 0.05), similar to *T*_2*b*_, as presented in Table 1. However, *P*_21_ and *P*_22_ were significantly influenced by the PDF amount (*p* < 0.05): *P*_21_ increased from 91.2% (without PDF added) to 94.7% (with 3.0% PDF added), then decreased to 91.0% when the level reached 4.0%, while *P*_22_ changed from 6.76% to 2.91%, then to 6.55%. This result indicates that the appropriate PDF amounts could inhibit the water transformation from immobilized water to free water in sample, and 3.0% PDF produced the optimal outcome. Protein denaturation and its structure change induced the patties’ water holding capacity to decrease during heating; therefore, a portion of immobilized water convert to free water and release from patties. While PDF could fix the converted and free water in the patties, which showed an increase in *P*_21_, there was a decrease in *P*_22_. However, when PDF exceeds 3.0%, the water absorption capacity of the fiber may be lower than the damage degree of the protein gel network, so there is a decrease in moisture migration inhibition efficiency (Table 1).

#### 3.1.4. Moisture Distribution

Pseudo-color images of magnetic resonance imaging (MRI) can visually exhibit the moisture distribution of the meat matrix foods. A color bar was used to scale the density of protons from water molecules, where blue represents the lower density and red represents the higher density of protons. The uniform degree of a single color in pseudo-color images represents the moisture distribution homogenization of patties [29].

**Figure 4:**
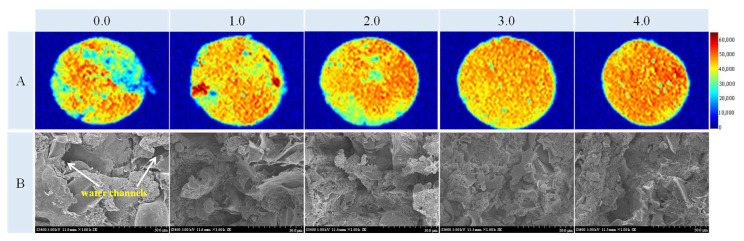
Influence of PDF on moisture distribution (**A**) and microstructure (**B**, 1000×) of fried chicken patty. Yellow arrows represent water channels. 0.0: control; 1.0: treatment with 1.0% PDF; 2.0: treatment with 2.0% PDF; 3.0: treatment with 3.0% PDF; 4.0: treatment with 4.0% PDF. PDF: potato dietary fiber.

Reddish image areas of the samples increased gradually with the increase in PDF (Figure 4A), implying that there were more water molecules in patties when PDF increased. Ullah et al. [30] also reported that the tofu gels’ pseudo-color images treated with okara dietary fiber were more reddish than the pure gel. The more PDF that is added, the more uniform the image (<3.0%) because there are more single orange and yellow areas in the images. In contrast, there was a large blue area in patties without PDF, as depicted in Figure 4A, likely because the meat–protein structure here is loose and has a large cavity. Thus, free water is difficult to trap in the network structure.

However, there was less homogenization when the addition of PDF exceeded 3.0% because some green holes could be visualized in the image. The more water molecules and uniformity in patties with the increase in PDF could be attributed to the fact that the PDF with water-binding and absorbing capacities decreases the exudation of water and more water can be retained in patties, maintaining the shape and increasing their weight (Figure 2). PDF can retain more water and improve the final quality of the patties.

#### 3.1.5. Color

The surface color parameters of lightness (L*), redness (a*), and yellowness (b*) of fried chicken patties formulated with different amounts of PDF are shown in Table 2. All of these parameters were significantly influenced by PDF (*p* < 0.05). The L*-value of the fried chicken patties was increased significantly as the content of PDF increased (*p* < 0.05) and maintained between 3.0% (61.2) and 4.0% (61.6) treatments. The increase in L*-value may be attributed to the addition of bright white PDF (CIE L* = 88.3 ± 0.07; a* = 1.55 ± 0.23; b* = 9.59 ± 0.08), which is insoluble and further lead to an increase in the light reflection intensity of the patties [31].

The a*-value of fried chicken patties with PDF increased significantly compared with the control except for the 4.0% treated group samples (*p* < 0.05). The a*-value of cooked patties is related to the undenatured myoglobin and the formation of globin hemochromogen [32], and also related to Maillard reaction, which predominates all other reactions in the color formation at the surface of fried patties [33]. It is speculated that this parameter may be associated with the difference in the resource and the incorporation methods of the addition of non-meat ingredients, besides the method of processing.

The b*-value showed a significant decreasing trend with the increase of PDF (*p* < 0.05). Zhao et al. [34] also reported a downward trend of b*-value in fat-reduced emulsified sausage formulated with the increase in the amount of regenerated cellulose fiber and speculated that the relative decrease in fat concentration led to the lower b*-value. Moreover, oil is uptaken on the crust and in the areas around the center layer just under the crust through the pores formed by water evaporation during the frying process in this study, indicating that the lesser the water evaporation, the lower the oil uptake [35]. Based on the results of moisture migration (Table 1) and moisture distribution (Figure 4A), the moisture loss of fried chicken patties with the addition of PDF was suppressed, which may lead to lower oil absorption and thus lower b*-value than the control group.

*∆E* indicates the total difference in color difference between treatments, and the larger the *∆E* value, the more obvious the color difference. Lenaerts et al. [36] concluded that color differences with *∆E* values below 1.5 are not visible, while those above 6 are clearly visible. As can be seen from Table 2, PDF increases the *∆E* value, i.e., the more PDF is added, the more obvious the color difference is, but the *∆E* values of all groups of samples are lower than 6, indicating that PDF does not cause consumers to perceive the color difference significantly.

#### 3.1.6. Texture Profile Analysis (TPA)

The influence of PDF on the textural properties (namely hardness, springiness, cohesiveness, gumminess, chewiness, and resilience) of chicken patties are presented in Table 3. The hardness, gumminess, and chewiness of patties with PDF increased and then decreased, with the highest value in the 3.0% PDF group. These parameters increased by 49.2%, 59.8%, and 74.6% compared with the control. Zhuang et al. [25] also reported that the hardness, gumminess, and chewiness of pork gel with 3.0% sugarcane dietary fiber increased by 67.2%, 62.7%, and 66.1% compared with the control. These results may be caused by the porous surface and dendritic structure of the dietary fiber which can be filled in meat processing products to play a synergistic role in forming a three-dimensional meat–protein network structure. Furthermore, the fibers absorb water and then swell in the meat matrix, pushing the ingredients in meat to bond tightly, thus increasing the hardness, gumminess, and chewiness of surimi gel [37].

The springiness of patties first increased and then decreased and there were maximum values in the samples with 2.0% and 3.0% PDF that were significantly higher than the control (Table 3). The cohesiveness increased and then decreased, with a maximum value in the sample with 2.0% PDF and significantly higher than the control (Table 3). The value of resilience had no significant changes among all sample groups (*p* > 0.05). Debusca et al. [38] also observed an increase and then decrease in springiness in surimi with wheat dietary fiber powder. However, Zhao et al. [34] stated that the springiness and cohesiveness of emulsified sausage all decreased with regenerated cellulose fiber increasing. These results suggested that the dietary fiber type may directly affect the textural properties of meat products.

### 3.2. Microstructure

The microstructure of chicken patties with or without PDF demonstrated significantly different appearances in Figure 4B. The microstructure of the patty was rough and uneven with some large “water channels” or porous cavities (marked with yellow arrows) that penetrated the meat–protein matrix. Zhuang et al. [39] stated that patty water-channel formation was induced by the water exuding from the internal gelation and turning into free water that can be quickly evaporated. Moreover, the appearance of water channels or even the interconnection of water channels may lead to the deterioration of the integrity of the meat–gel network, as represented by the mechanism diagram in the effect of sugarcane insoluble dietary fiber on pork gel [39].

The microstructure of the meat–protein matrix with PDF was uniform and dense, with smaller or even nonexistent water channels compared with the control, especially in the sample with 3.0% PDF (Figure 4B). This implies that PDF could absorb water and trap it in the gel network to prevent exudation while filling the gaps of the meat–protein–gel network to form a denser network structure than the control [9]. The improvement in the microstructure of chicken patties is also reflected in the increase in textural properties of the meat–protein matrix (Table 3). This is consistent with the result of Shi et al. [40], who reported that the relatively compact structure could withstand mechanical forces, thus enhancing the mechanical properties.

However, there were more pores again when the PDF reached 4.0%, and the cavities induced by the self-aggregation of fibers at a high amount may be so large that they destroy the continuity of the meat–protein matrix [9]. Furthermore, the water availability of the protein is reduced by fibers’ excessive water absorption, which may also disrupt the meat–protein–gel network [38]. The disruption in the meat–protein–gel network from a high PDF amount may lead to more water diffusing toward the crust and textural properties weakening compared with a low PDF sample (Table 3). However, there were still more water molecules in the sample with 4.0% PDF (Figure 4A), confirming the strong water absorption of PDF. PDF may improve the network structure of the meat matrix and improve the meat product quality by trapping the ingredients within it.

### 3.3. Thermal Stability

**Figure 5:**
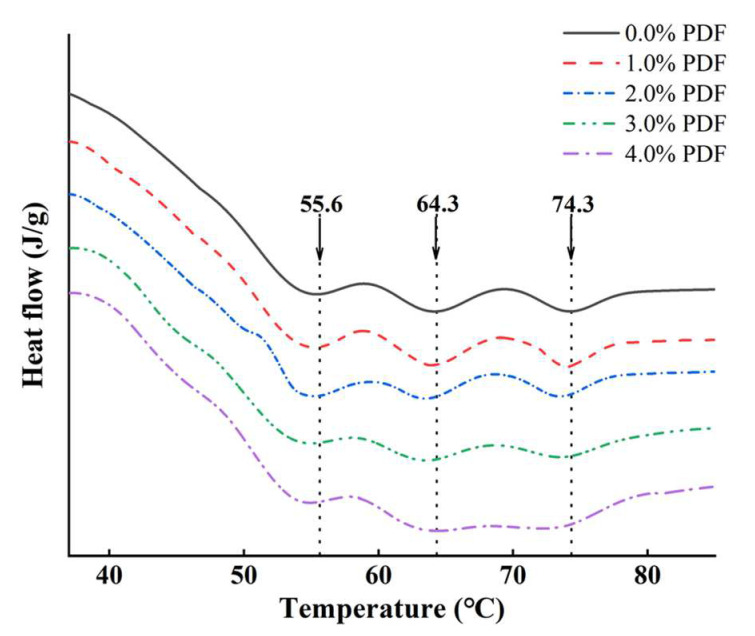
Influence of PDF on the protein denaturation temperature of chicken patty. PDF: potato dietary fiber.

Differential scanning calorimetry (DSC) is considered as an important index for analyzing the thermal stability of meat products and can be expressed in terms of *T*_max_ and *ΔH*. In this study, the denaturation temperature (*T*_max_) and enthalpy (*ΔH*) of the samples were monitored by DSC, as summarized in Figure 5 and Table 4. Typical transition temperatures of all samples whether or not PDF was added ranged from 53.0 to 55.0 °C for myosin (*T*_max1_), 62.0 to 64.0 °C for connective tissue (together with sarcoplasmic proteins) (*T*_max2_), and 72.0 to 76.0 °C for actin (*T*_max3_) [41]. In addition, *ΔH*_1_, *ΔH*_2_, and *ΔH*_3_ were the corresponding enthalpy values.

As shown in Table 4, the transition temperatures of the samples with different levels of PDF were changed compared with the control samples, but these values fluctuated in a small region. *ΔH*_1_, *ΔH*_2_, and *ΔH*_3_ were not significantly influenced by PDF (*p* > 0.05). Debusca et al. [38] stated that the addition of wheat dietary fiber does not interfere with normal denaturation and cross-linking of surimi pastes myosin or actin. The discrepancy between our results and this conclusion may be due to the fluctuations in other components formulated in the chicken patties due to the fiber being incorporated into the formulations in our present study, while the above study implied silicon dioxide (SiO_2_) as inert filler to control the same content of all other components except fiber. As stated by Choi et al. [42], heat treatment was the main factor that resulted in the denaturation of proteins. The fiber is only physically confined in the protein–gel network and has no direct cross-linking with proteins; the fiber–protein interaction is mediated by water, which can be clearly observed in paraffin sections by light microscopy, which was reported by Zhuang et al. [25]. Anyway, the addition of fiber cannot interfere with the denaturation of proteins, but the fiber can fix a large amount of water on its surface owing to its water-absorbing and binding properties, and the water-absorbing fibers are embedded in the meat–protein–gel matrix. Fiber changes the structure of proteins by water, affecting the interaction between proteins, improving the quality of the gel, and thus improving the gelling properties of meat protein [38]. Moreover, the previous study has reported that the dimensional change is mainly due to the denaturation of proteins [23], while the result of thermal stability in chicken patties demonstrated that the dimensional change of fried chicken patties by the addition of fiber (shown in Figure 2) does not correlate with the denaturation of proteins and is mainly reflected in its filling effect and the improvement of separation in water and fat induced by the protein denaturation.

### 3.4. Sensory Properties

Influence of PDF on the sensory properties of fried chicken patty is shown in Table 5. There was no difference in color scores among samples with or without PDF (*p* > 0.05), indicating that the PDF did not cause visually significant color differences, which is consistent with the result of color (Table 2). The flavor of fried chicken patties did not change significantly when PDF was added at 3% or less (*p* > 0.05), but a slight decrease in flavor occurred when the PDF added rose further, probably due to the high amount of PDF added, which somewhat masked the original frying flavor of the patties.

The juiciness of the patties showed a tendency to increase and then decrease with PDF increasing, which was attributed to the strong water absorption and water stabilization ability of PDF and less water loss during cooking process (Figure 3). Zhao et al. [34] also reported that the regenerated cellulose fiber increases the juiciness scores of fat-reduced emulsified sausage. Although MRI maps showed the highest moisture content in sample with 4.0% PDF, the decrease in juiciness may be due to the high dry matter content in the chicken patties resulting from the excess dispersion of PDF, which produced a coarse mouthfeel and a relative decrease in juiciness. The texture score showed a trend of increasing and then decreasing with the increase of PDF, and the highest score was obtained in sample with 2.0% PDF. The results of texture properties showed that PDF had a positive effect on the hardness, gumminess, and chewiness of patties (Table 3), so the texture score was higher than the control when the PDF was below 2%, and when the PDF content further increased, the excessive hard mechanical properties led to a poorer chewy texture. The overall acceptability results showed that PDF did not have a significant negative effect on the sensory scores of chicken patties at additions of 3% or less.

### 3.5. Proximate Composition

The proximate composition of fried chicken patty was significantly influenced by PDF (Table 6). The moisture content was increased significantly with PDF increasing (*p* < 0.05), and there was a maximum value in sample with 4.0% PDF. This result indicated that PDF allows more water to be retained in chicken patty, which was in accordance with the result of moisture distribution (Figure 4A). This is because the PDF has a strong water-binding capacity and improves water- and fat-binding properties of patties [34]. The fat content decreased significantly with increasing PDF (*p* < 0.05), which could explain the decrease of b* (Table 2). Previous studies [22,43,44] also found the same result, in which all of wheat sprout, washed cashew apple fiber, and *Algelica keiskei* Koidz fiber could reduce the fat content of chicken patty. There was no significant difference in protein content of fried chicken patty (*p* > 0.05); Zhao et al. [34] and Guedes-Oliveira et al. [43] also reported that regenerated cellulose fiber did not influence the protein content of fat-reduced emulsified sausage and chicken patties. The increase in the content of ash and carbohydrates was due to the addition of PDF, which is mostly composed of carbohydrate polymers [45]. The energy value was decreased significantly with PDF increasing (*p* < 0.05), and there was a minimum value in chicken patty with 4.0% PDF, which indicated that PDF can not only improve the quality of fried chicken patty, but also can decrease energy value and improve nutrient properties.

## 4. Conclusions

The effect of PDF on the quality, microstructure, and thermal stability of chicken patties was evaluated. PDF improved the quality of chicken patty, including inhibiting the dimensional change, improving the water- and fat-holding properties, textural properties, and promoting the transformation of free water to immobilized water. PDF promoted a more homogeneous and dense meat–protein network structure to be formed due to its filling and dehydration effects. Moreover, the results of the thermal stability of patties showed that PDF acts mainly through its filling effect and suppression of water and fat separation induced by protein denaturation during the heating process and does not interfere with the denaturation of proteins. At the same time, the PDF addition of less than 3.0% did not have a significant negative impact on the sensory score, while the nutritional quality was improved. In conclusion, moderate addition of PDF can enhance nutrient composition while improving the quality of processed meat products, which may offer more attractive possibilities for the healthier processed meat processing industry.

## Figures and Tables

**Table 1:** Influence of PDF on moisture migration of fried chicken patty.

PDF (%)	*T*_2_ (ms)			*P*_2_ (%)		
	*T*_2b_	*T*_21_	*T*_22_	*P*_2b_	*P*_21_	*P*_22_
0.0	0.69 ± 0.02	42.5 ± 1.7 ^a^	349 ± 14 ^a^	2.08 ± 0.38	91.2 ± 0.3 ^d^	6.76 ± 0.49 ^a^
1.0	0.79 ± 0.07	37.9 ± 1.5 ^b^	318 ± 13 ^b^	2.17 ± 0.22	92.3 ± 0.6 ^c^	5.54 ± 0.57 ^b^
2.0	0.78 ± 0.06	35.3 ± 1.4 ^b^	318 ± 13 ^b^	2.26 ± 0.21	93.5 ± 0.5 ^b^	4.23 ± 0.50 ^c^
3.0	0.72 ± 0.04	30.1 ± 1.2 ^c^	297 ± 12 ^b^	2.41 ± 0.01	94.7 ± 0.2 ^a^	2.91 ± 0.22 ^d^
4.0	0.80 ± 0.06	28.7 ± 1.1 ^c^	258 ± 10 ^c^	2.41 ± 0.10	91.0 ± 1.0 ^d^	6.55 ± 0.87 ^ab^

Values represent mean ± SE of at least triplicate determinations. *T*_2b_: bound water; *T*_21_: immobilized water; *T*_22_: free water; *P*_2b_: the percentage of bound water; *P*_21_: the percentage of immobilized water; *P*_22_: the percentage of free water; PDF: potato dietary fiber. Different letters (a to d) in a column indicate significant differences (*p* < 0.05) between treatments with different amounts of potato dietary fiber.

**Table 2:** Influence of PDF on the color of fried chicken patty.

PDF (%)	L*	a*	b*	*∆E*
0.0	58.3 ± 0.7 ^d^	14.8 ± 0.5 ^c^	38.4 ± 0.7 ^a^	-
1.0	58.9 ± 0.4 ^c^	15.4 ± 0.5 ^b^	37.6 ± 0.5 ^b^	1.38 ± 0.38 ^c^
2.0	59.8 ± 0.6 ^b^	15.7 ± 0.4 ^ab^	36.0 ± 0.6 ^c^	3.07 ± 0.54 ^b^
3.0	61.2 ± 0.4 ^a^	15.9 ± 0.6 ^a^	35.9 ± 1.2 ^c^	4.11 ± 1.06 ^a^
4.0	61.6 ± 0.5 ^a^	14.1 ± 0.4 ^d^	35.1 ± 0.2 ^d^	4.71 ± 0.37 ^a^

Values represent mean ± SE of at least triplicate determinations. PDF: potato dietary fiber. Different letters (a to d) in a column indicate significant differences (*p* < 0.05) between treatments with different amounts of potato dietary fiber.

**Table 3:** Influence of PDF on the textural properties of fried chicken patty.

PDF (%)	Texture Properties					
	Hardness (g)	Springiness (%)	Cohesiveness (%)	Gumminess (g)	Chewiness (g.s)	Resilience (%)
0.0	1636 ± 34 ^c^	0.38 ± 0.03 ^b^	0.45 ± 0.03 ^b^	736 ± 66 ^c^	283 ± 46 ^c^	0.18 ± 0.02
1.0	1805 ± 159 ^c^	0.42 ± 0.01 ^ab^	0.47 ± 0.02 ^b^	842 ± 88 ^c^	350 ± 37 ^b^	0.18 ± 0.01
2.0	2085 ± 142 ^b^	0.42 ± 0.01 ^a^	0.51 ± 0.02 ^a^	1052 ± 25 ^b^	470 ± 22 ^a^	0.19 ± 0.01
3.0	2441 ± 93 ^a^	0.42 ± 0.01 ^a^	0.48 ± 0.01 ^ab^	1176 ± 29 ^a^	494 ± 24 ^a^	0.19 ± 0.01
4.0	2421 ± 165 ^a^	0.41 ± 0.01 ^ab^	0.46 ± 0.01 ^b^	1118 ± 73 ^ab^	455 ± 41 ^a^	0.18 ± 0.01

Values represent mean ± SE of at least triplicate determinations. PDF: potato dietary fiber. Different letters (a to c) in a column indicate significant differences (*p* < 0.05) between treatments with different amounts of potato dietary fiber.

**Table 4:** Influence of PDF on the thermal properties of proteins in chicken patty.

PDF (%)	Thermal Properties					
	*T*_max1_ (°C)	*T*_max2_ (°C)	*T*_max3_ (°C)	*ΔH*_1_ (J/g)	*ΔH*_2_ (J/g)	*ΔH*_3_ (J/g)
0.0	55.6 ± 0.3 ^a^	64.3 ± 0.1	74.3 ± 0.2 ^a^	0.503 ± 0.011	0.291 ± 0.018	0.477 ± 0.005
1.0	55.5 ± 0.3 ^ab^	64.0 ± 0.4	74.3 ± 0.2 ^a^	0.498 ± 0.013	0.289 ± 0.015	0.480 ± 0.005
2.0	55.4 ± 0.3 ^ab^	64.0 ± 0.2	73.6 ± 0.4 ^b^	0.491 ± 0.012	0.286 ± 0.011	0.475 ± 0.012
3.0	55.4 ± 0.4 ^ab^	63.8 ± 0.4	74.2 ± 0.3 ^ab^	0.487 ± 0.009	0.281 ± 0.016	0.474 ± 0.005
4.0	54.9 ± 0.3 ^b^	64.0 ± 0.1	73.6 ± 0.5 ^b^	0.488 ± 0.008	0.280 ± 0.012	0.467 ± 0.011

Values represent mean ± SE of at least triplicate determinations. *T*_max1_: the temperature of thermal denaturation of myosin; *T*_max2_: the temperature of thermal denaturation of connective tissue and sarcoplasmic proteins; *T*_max3_: the temperature of thermal denaturation of actin; *ΔH*_1_: the amount of energy to thermal denaturation of myosin; *ΔH*_2_: the amount of energy to thermal denaturation of connective tissue and sarcoplasmic proteins; *ΔH*_3_: the amount of energy to thermal denaturation of actin. PDF: potato dietary fiber. Different letters (a to b) in a column indicate significant differences (*p* < 0.05) between treatments with different amounts of potato dietary fiber.

**Table 5:** Influence of PDF on the thermal properties in chicken patty.

PDF (%)	Color	Flavor	Juiciness	Texture	Overall Acceptability
0.0	5.43 ± 0.52	5.33 ± 0.76 ^a^	5.06 ± 0.54 ^ab^	5.27 ± 0.38 ^ab^	5.75 ± 0.72 ^a^
1.0	5.36 ± 0.78	5.38 ± 0.66 ^a^	5.22 ± 0.56 ^ab^	5.37 ± 0.40 ^ab^	5.51 ± 0.48 ^a^
2.0	5.31 ± 0.74	5.18 ± 0.79 ^ab^	5.43 ± 0.62 ^a^	5.53 ± 0.59 ^a^	5.62 ± 0.64 ^a^
3.0	5.60 ± 0.46	5.11 ± 0.61 ^ab^	5.50 ± 0.44 ^a^	4.99 ± 0.22 ^bc^	5.39 ± 0.32 ^ab^
4.0	5.31 ± 0.51	4.55 ± 0.50 ^b^	4.80 ± 0.58 ^b^	4.73 ± 0.79 ^c^	4.94 ± 0.57 ^b^

Values represent mean ± SE of at least triplicate determinations. PDF: potato dietary fiber. Different letters (a to c) in a column indicate significant differences (*p* < 0.05) between treatments with different amounts of potato dietary fiber.

**Table 6:** Influence of PDF on the proximate composition in chicken patty.

PDF (%)	Moisture (%)	Fat (%)	Protein (%)	Ash (%)	Carbohydrates (%)	Energy Value (kal/100 g)
0.0	60.8 ± 0.3 ^c^	16.7 ± 0.8 ^a^	17.2 ± 0.5	2.59 ± 0.06 ^b^	2.74 ± 0.54 ^b^	230 ± 3 ^a^
1.0	61.5 ± 0.5 ^bc^	15.8 ± 0.4 ^ab^	16.1 ± 0.4	2.84 ± 0.10 ^a^	3.73 ± 0.40 ^ab^	221 ± 3 ^b^
2.0	62.6 ± 0.2 ^ab^	14.9 ± 0.8 ^bc^	15.8 ± 0.7	2.83 ± 0.01 ^a^	3.87 ± 0.56 ^ab^	212 ± 5 ^c^
3.0	62.9 ± 1.5 ^a^	14.6 ± 0.3 ^c^	15.9 ± 1.2	2.81 ± 0.04 ^a^	3.69 ± 0.16 ^ab^	210 ± 7 ^c^
4.0	63.2 ± 0.1 ^a^	13.9 ± 0.4 ^c^	15.6 ± 1.5	2.63 ± 0.05 ^b^	4.64 ± 1.18 ^a^	206 ± 1 ^c^

Values represent mean ± SE of at least triplicate determinations. PDF: potato dietary fiber. Different letters (a to c) in a column indicate significant differences (*p* < 0.05) between treatments with different amounts of potato dietary fiber.

## Data Availability

The data presented in this study are available in the article.
